# Lineage diversification and historical demography of a montane bird *Garrulax elliotii *- implications for the Pleistocene evolutionary history of the eastern Himalayas

**DOI:** 10.1186/1471-2148-11-174

**Published:** 2011-06-21

**Authors:** Yanhua Qu, Xu Luo, Ruiying Zhang, Gang Song, Fasheng Zou, Fumin Lei

**Affiliations:** 1 Key Laboratory of Zoological Systematics and Evolution, Institute of Zoology, Chinese Academy of Sciences, Beijing 100101, China; 2 Faculty of Conservation Biology, Southwest Forestry University, Kunming, 650224, China; 3 South China Institute of Endangered Animals, Guangzhou 510260, China

**Keywords:** Pleistocene glaciations, sky islands, diversification, eco-subregion, multiple refugia, genetic admixture

## Abstract

**Background:**

Pleistocene climate fluctuations have shaped the patterns of genetic diversity observed in many extant species. In montane habitats, species' ranges may have expanded and contracted along an altitudinal gradient in response to environmental fluctuations leading to alternating periods of genetic isolation and connectivity. Because species' responses to climate change are influenced by interactions between species-specific characteristics and local topography, diversification pattern differs between species and locations. The eastern Himalayas is one of the world's most prominent mountain ranges. Its complex topography and environmental heterogeneity present an ideal system in which to study how climatic changes during Pleistocene have influenced species distributions, genetic diversification, and demography. The Elliot's laughing thrush (*Garrulax elliotii*) is largely restricted to high-elevation shrublands in eastern Himalayas. We used mitochondrial DNA and microsatellites to investigate how genetic diversity in this species was affected by Pleistocene glaciations.

**Results:**

Mitochondrial data detected two partially sympatric north-eastern and southern lineages. Microsatellite data, however, identified three distinct lineages congruent with the geographically separated southern, northern and eastern eco-subregions of the eastern Himalayas. Geographic breaks occur in steep mountains and deep valleys of the Kangding-Muli-Baoxin Divide. Divergence time estimates and coalescent simulations indicate that lineage diversification occurred on two different geographic and temporal scales; recent divergence, associated with geographic isolation into individual subregions, and historical divergence, associated with displacement into multiple refugia. Despite long-term isolation, genetic admixture among these subregional populations was observed, indicating historic periods of connectivity. The demographic history of *Garrulax elliotii *shows continuous population growth since late Pleistocene (about 0.125 mya).

**Conclusion:**

While altitude-associated isolation is typical of many species in other montane regions, our results suggest that eco-subregions in the eastern Himalayas exhibiting island-like characteristics appear to have determined the diversification of *Garrulax elliotii*. During the Pleistocene, these populations became isolated on subregions during interglacial periods but were connected when these expanded to low altitude during cooler periods. The resultant genetic admixture of lineages might obscure pattern of genetic variation. Our results provide new insights into sky island diversification in a previously unstudied region, and further demonstrate that Pleistocene climatic changes can have profound effects on lineage diversification and demography in montane species.

## Background

Climatic oscillations over the past few million years have had profound effects on the demography of, and patterns and levels of genetic diversity in, many extant species [1-4]. Temperate taxa inhabiting alpine habitats often shift their ranges along an altitudinal gradient in response to climatic changes. Therefore, populations on different mountains may experience alternating periods of isolation and connectivity [2-5]. Periods of isolation may result in genetic divergence among populations, whereas periods of connectivity allow for dispersal and gene flow [1,2]. Because species' responses to climate change are influenced by interactive factors including ecology, demography and local topography, the pattern and tempo of diversification may differ between taxa and from place to place [1-4]. For example, the amount of time that populations are isolated and their effective population size will determine whether they sort into independent evolutionary lineages, whereas the level of divergence and ecological characteristics of the species often influence whether they remain distinct or merge back into a common gene pool upon secondary contact [2,6]. Studies of genetic diversification in alpine regions are currently limited to a few areas [7-14]. Additional research in other alpine regions is therefore required to confirm the generality of existing hypotheses on how Pleistocene climatic change affected species diversification and demography.

The eastern Himalayan mountain range, which has perhaps the most complex topography on Earth, offers an unusual opportunity to study interactions among geography, climatic changes and diversification. These mountains rose rapidly with the uplift of the Tibetan Plateau during the past three million years [15-18]. Mountains are characterized as a series of parallel alpine ranges climbing to altitudes over 5 000 m above sea level (m.a.s.l.) with the differences in altitude from valley to mountaintops often exceeding 2 000 m.a.s.l. This broadly altitudinal range has created dramatic ecological stratification and resulted in geographic isolation for many taxa [19-21]. Tremendous variations in altitude, climate and vegetation may have created an archipelago of high elevation sky islands [22,23]. The Elliot's laughing thrush (*Garrulax elliotii*) is endemic to the eastern Himalayan region [23-26], where it generally occupies shrubland habitats at altitudes from 2 000 to 4 000 m.a.s.l. Since the montane habitat in this region is divided by deep valleys into distinct eco-subregions or clusters of mountains [19,20], it is plausible that these geographic barriers restrict gene flow between *G. elliotii *populations thus contributing to genetic diversity in this bird.

High altitude parts of the eastern Himalayas were subject to repeated glaciations during the Pleistocene [27]. These glacial cycles and accompanying climate changes probably had a large influence on species' distribution and demography [28,29]. The range of *G. elliotii *is predicted to have expanded and contracted along an altitudinal gradient resulting in historic periods of connectivity and isolation. Given the potential for repeated contacts between populations on different mountains, it is possible that populations merged into a single gene pool during historic periods of connectivity [1,2,6]. However, many studies in other montane regions suggest that gene flow among populations remained restricted during the Pleistocene, leading to each population remaining a distinct evolutionary lineage [8-14].

We used mitochondrial DNA and microsatellites to address the effects of Pleistocene climatic changes on genetic diversification in the eastern Himalayas. We first evaluate whether Elliot's laughing thrush from each eco-subregion or cluster of mountains comprises a distinct evolutionary lineage. Second, we investigate the contribution of geographic and environmental barriers to *G. elliotii*'s distribution. Third, we test whether the timing of diversification within *G. elliotii *is consistent with climatic shifts induced by Pleistocene glacial cycles. Fourth, we use coalescent simulations to test whether populations from different mountains have descended from a single ancestor (single refugium), or, alternatively, remained isolated on individual mountains during down-slope expansions (multiple refugia). Finally, given that the range of *G. elliotii *is predicted to have expanded and contracted in response to Pleistocene climatic fluctuations, we also examine historical demography of this species.

## Materials and methods

### Study area and sampling sites

**Figure 1:**
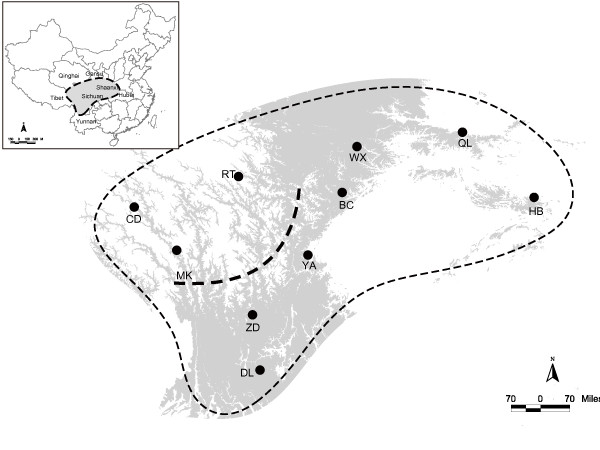
**Topographic map of the eastern Himalayan region including all elevations between 2 000 and 4 000 m.a.s.l.; the altitudinal range of Elliot's laughing thrush (*Garrulax elliotii*)**. The species' range is indicated by the thin dashed line as in [110]. Sampling locations are indicated by the abbreviation of each population's name (Table 1). The bold dashed line represents the boundary between recognized ecological subregions of the eastern Himalayan mountain range [20]. Insert: The shaded area represents the study region.

The eastern Himalayan mountain region occupies western and north-western Yunnan, western Sichuan, south-eastern Tibet, southern Qinghai and south-western Gansu. Steep mountain ranges and major rivers in deep valleys divide this region into several distinct eco-subregions [19,20] (see Figure 1). Tang [24] and Zhang [30] described diverse floristic constitution of these subregions. The northern subregion, which is comprised a part of the Tibetan plateau, is dominated by a plateau cold and dry climatic condition [24-27,30]. The vegetation type is characterized by alpine meadows and grasslands. This northern subregion is considered as 'Plateau avifauna' with the endemic avian species as *Pseudopodoces humilis, Montifringilla adamsi, Pyrgilauda ruficollis, Urocynchramus pylzowi *and *Kozlowia roborowskii *[24,31,32]. The southern subregion, on the other hand, is influenced by much warmer and wetter climatic condition, and the vegetation is characterized by tropical, or subtropical, broadleaved forests with a mixture of evergreen and deciduous species. This subregion is represented by Himalaya-Hengduanshan Mountain avifauna with many endemics babbles (Timallinae) and pheasants [24-26]. The eastern subregion consists of a cluster of mountains including of Qinling, Min, Mang and Daba mountains. The vegetation is dominated by temperate evergreen coniferous and broadleaved mixed forests. Endemic avian species include *Chrysolophus pictus, Spizixos semitorques, Garrulax sukatschewi, G. davidi, Yuhina diademata, Paradoxornis przewalskii, P. paradoxus *and *Parus davidi *[24-26,31-33]. Subregion-specific species have also been observed for other taxa, such as plants [34], ants [35], fishes [36], reptiles [37] and mammals [38].

These three subregions are further divided into thirteen biogeographic sectors corresponding to clusters of mountains [20]. The sites from which samples of *G. elliotii *were obtained are shown in Figure 1 and Table 1. These sample sites were distributed across the range of the species, and grouped into ten populations according to the biogeographic sectors described in Li [20].

**Table 1:** Nucleotide polymorphism in each population; S, number of segregating sites; Nhap, number of haplotypes; Hd, haplotype diversity; π, nucleotide diversity

Subregion division	**Biogeographic sectors in Li **[20]	Sampling locations	*n*	S	Nhap	Hd	π
Southern subregion	Southwest Yunnan mountains	DL	11	39	10	0.982	0.00338
	Yulong mountains	ZD	14	58	13	0.989	0.00385
Eastern	Motianling mountains	WX	7	27	5	0.905	0.00279
subregion	Qinlin mountains	QL	5	26	5	1	0.00318
	Shenlongjia mountains	HB	5	33	5	1	0.00338
	Minshan mountains	BC	7	28	7	1	0.00298
	Wuxia mountains	YA	8	7	34	0.964	0.00355
Northern	Gongga mountains	MK	14	45	10	0.923	0.00297
subregion	Ningjing mountains	CD	7	55	6	0.952	0.00451
	North Shaluli mountains	RT	2	20	2	1	0.00496

### Mitochondrial DNA amplification and sequencing

DNA was extracted from tissue samples using the DNeasy Tissue kit (QIAGEN). The cytochrome *b *(cyt-*b*), cytochrome *c *oxidase subunit I and II (COI and COII), NADH dehydrogenase subunit 2 and 6 (ND_2 _and ND_6_), ATP synthetase subunit 8 (ATP_8_) and control region (CR) genes were amplified via the polymerase chain reaction (PCR) [39-44]. The same primers were used for amplification and sequencing reactions. Methods of purification and sequencing were as described in Qu et al. [45]. All sequences are accessible at GenBank (Accession nos: HM601543-602022).

### Microsatellite genotyping

PCR amplifications were achieved in a 10 ul reaction volume containing 10-30 ng DNA, 0.5 U Taq DNA polymerase, 0.2 nmol dNTPs, 1-3 nmol MgCl_2 _and 5 pmol primers. These loci were originally developed for the following passerine species: *Hirundo rustica *(HrU_5_-A) [46], *Loxia scotica *(LOX_1_) [47], *Catharus ustulatus *(Cum_02_, Cum_10_, Cum_28 _and Cum_32_) [48], *Poecile atricapillus *(Pat_43_) [49], *Phylloscopus occipitalis *(Pocc_6 _and Pocc_8_) [50] and *Dendroica petechia *(Dpu_16_) [51]. Original PCR conditions were optimized with variable MgCl_2_, template DNA concentrations and annealing temperatures. Microsatellites were amplified with fluorescently labelled forward primers (FAM and HEX dyes). Fragment lengths were analyzed with the internal size marker GENESCAN-500 ROX (Applied Biosystems), and scored with GENEMARKER 3.7 (SoftGenetics).

### Genetic polymorphism

For mitochondrial data, we calculated the number of segregating sites, haplotype diversity and nucleotide diversity for each population and all populations combined using DnaSP 4.0 [52]. McDonald & Kreitman's [53] test was used to examine the selective neutrality of the mitochondrial DNA protein-coding fragments. Two additional neutrality tests, Fu's *F*_S _[54] and Fu & Li's [55]*D *were used to detect departures from the mutation-drift equilibrium that would be indicative of changes in historical demography and natural selection. All three tests were implemented in DnaSP.

For microsatellite data, parameters such as the number of alleles per locus (*N*_A_), average allelic richness (*A*_R_), observed heterozygosity (*H*_O_), heterozygosity expected from the Hardy-Weinberg assumption (*H*_E_) and exact tests of linkage disequilibrium (LD) between pairs of loci for each population were calculated with ARLEQUIN 3.5 [56] and FSTAT 2.9.4 [57].

### Phylogeography and population structure

The maximum-likelihood algorithm implemented in PHYML 2.4.4 [58] was used to reconstruct phylogenetic relationships among mitochondrial haplotypes. Based on the Akaike Information Criterion (AIC) [59], we used the model GTR+I+G as suggested by Modeltest 3.7 [60]. We set the proportion of invariant sites and the shape of Gamma distribution as 0.43 and 1.048, respectively (estimated by Modeltest). The base frequency and ratio of transition/transversion were optimized by the maximum-likelihood criterion in PHYML. Outgroups were the black-faced laughing thrush (*G. affinis*), exquisite laughing thrush (*G. formosus*) and streaked laughing thrush (*G. lineatus*), which are considered closely related to *G. elliotii *[61].

We used two methods to identify genetically distinct groups among microsatellite genotypes. First, genetic relationships between individual samples were quantified using Nei's standard genetic distance in GENETIX 4.05 [62]. The resultant matrix was then converted into a dendrogram using the Neighbour-Joining algorithm [63] provided with PHYLIP 3.5 [64] and graphically displayed with TREEVIEW 1.6 [65]. Second, we used STRUCTURE 2.3.2 [66] with a burn-in of 5 × 10^7 ^and 5 × 10^8 ^iterations without prior population information and following the admixture model. We conducted ten replicate runs for each value of *K *(most likely number of populations) from 1 to 10. The most likely *K *was identified using the maximal values of Pr(*X*/*K*), typically used for STRUCTURE analysis [66] and Δ*K *based on the rate of change in the posterior probability of data between successive *K*-values [67].

### Landscape genetics and environmental variables correlation analyses

Suitable habitats for *G. elliotii *were identified in order to investigate whether intraspecific lineages were separated by areas of unsuitable environmental conditions. The habitat-mapping model was developed in ARCGIS 9.0 (Environmental Systems Research Institute) using vegetation, topographic layers and the known distribution range of *G. elliotii*. Shrublands were identified as suitable vegetation types [24-26]. We consider an area suitable if these vegetation types are intersected by suitable altitude distribution (2 000 to 4 000 m.a.s.l.). These constructed areas were then projected onto the current distribution of *G. elliotii *to generate a map of suitable habitats. Gaps between patches of suitable habitat were considered habitat gaps. GENELAND 1.0.5 [68], a computer program in the R-PACKAGE [69], was implemented to verify our definition of *G. elliotii *populations and locate areas of genetic discontinuity. GENELAND used microsatellite genotype data together with geographic information (the locations from which individuals were sampled) to estimate population structure. Areas of genetic discontinuity were detected as geographic areas with low posterior probability of membership. Following the approach of Coulon et al. [70], we allowed *K *to vary (from 1 to 10) and inferred the most probable *K *using five replicate runs with 5 × 10^5 ^Markov chain Monte Carlo (MCMC) iterations. For these analyses the maximum rate of the Poisson process was fixed at 100 with no uncertainty in the spatial coordinates, and the maximum number of nuclei in the Poisson-Voronoi tessellation was fixed at 300. The Dirichlet model was used to estimate allele frequencies.

Additionally, a distance-based redundancy analysis [71,72] was used to examine whether environmental factors might explain genetic diversification among populations. In the case of mitochondrial data, we used ten locations as spatial units and the uncorrected p distance and pairwise Φ_ST _as the measures of genetic differentiation. In the case of microsatellite data, genetic distance was measured as the *F*_ST _and Nei's *D*s. An array of predictor variables was grouped into six sets: (i) coordinate (latitude and longitude); (ii) vegetation (alpine steppe; Tibet alpine meadow & shrublands; subalpine grassland & shrublands; temperate grasslands & shrublands and subtropical shrublands); (iii) subregion (southern; northern and eastern); (iv) elevation; (v) temperature (mean annual temperature; mean January temperature and mean July temperature); (vi) mean annual rainfall. All predictor variables except vegetation and subregion were continuous, therefore these were treated as categorical variables with two states; 1 if a sample was located in a given vegetation type or subregion, and 0 if it was located in another vegetation type or subregion. Thus, each vegetation type or subregion was regarded as a vector corresponding to five vegetation types and three subregions, respectively. Vegetation or subregion was analyzed as a set, i.e. these variables were combined in a single test.

Environmental variables were taken from the WWF Terrestrial Ecoregion and WorldClimate (http://www.worldclimate.com) databases. Subregions were defined according to Li [20]. In order to identify variables correlated with genetic distance, individual sets of predictor variables were analyzed with the marginal test in DISTLM 5.0 [73]. The *P *values in these analyses were obtained using 9 999 simultaneous permutations of the rows and columns of the distance matrix. To examine which subset of predictor variables provided the best model explaining genetic differentiations among *G. elliotii *populations, the forward selection procedure in the program DISTLM forward [74] was performed on all sets of variables. The forward selection procedure consists of sequential tests, fitting each set of variables one at a time, conditional on the variables already included in the model. To examine whether the tested variables were correlated, output files also included information on the correlations among all pairs of explanatory variables. This provided a further check on potential multi-collinearity issue. As in the previous analysis, *P *values were obtained using 9 999 permutations of the rows and columns of the multivariate residual matrix under the reduced model.

### Divergence time estimate

In order to overcome the limitations of conventional estimates of divergence time based on *F*_ST _values (e.g. estimation of divergence time assuming isolation without gene flow) [75-77], we employed a coalescent-based MCMC simulation to estimate the divergence times for the main mitochondrial lineages in the program IM [78,79]. As recommended by Hey & Nielsen [78] we first performed multiple runs, with an increasing number of steps and using wide priors and heating schemes to ensure that the complete posterior distribution could be obtained. We finally performed four independent runs of 5 × 10^8 ^steps with a burn-in of 5 × 10^7 ^steps and a linear heating scheme (g1 = 0.05). IM was run under the HKY substitution model (estimated by Modeltest 3.7). Convergence on stationary distributions was assessed by monitoring the similarity of posterior distributions from independent runs and by assessing the autocorrelation parameter values over these runs [80]. The peaks of the resulting posterior distributions were taken as estimates of parameters [81]. Credibility intervals were recorded as the 90% highest posterior density (HPD). Because no fossil data were available to calibrate the mutation rate, we assumed a conventional molecular clock for the avian mitochondrial DNA cytochrome *b *gene (1 × 10^-8 ^per site per year) [82,83]. The mutation rate was modulated by multiplying the ratio of the average distance for the combined sequences versus that for cyt *b *alone to deduce the mutation rate for all fragments combined. We used this modified mutation rate to convert the divergence time estimate into calendar years.

For microsatellite data, timing of population splits was estimated using the IMa2 [81] with burn-ins of 5 × 10^7 ^and 5 × 10^8 ^iterations under the SSM model with initial short runs to provide prior estimates. A geometric heating scheme was employed and the program run four times with different random seed numbers to ensure convergence of parameter estimates. The IMa2 output included mean values of the parameters for two sets after the burn-in period, representing the first and second half of the total generations of the post burn-in run. We ran our analyses until the peak location set values were equal for both sets. This, along with the high effective sample size and low autocorrelation estimate, suggested the Markov chains reached convergence after a sufficiently long burn-in period and were sampled from the appropriate likelihood space. A generation time of 1 year [26] and a mutation rate of 10^-5 ^to 10^-4 ^per generation for birds [84-89] were used to convert divergence time into calendar years. Given the uncertainty in mutation rates, the resultant estimates should be interpreted with caution.

### Historical biogeography

To determine whether populations remained isolated in individual habitats (multiple refugia), or merged into a single gene pool (single refugium) during down-slope dispersal, we used coalescent simulations in MESQUITE 2.5 [90] to test three phylogeographic models of diversification. The first hypothesis posited that all populations derive from a single source population (single-refugium hypothesis). The second hypothesis postulated that populations were isolated into southern and north-eastern refugia (two-refugia hypothesis). The geographic structure was inferred from mitochondrial data. A third, three-refugia hypothesis, which was consistent with three-subregion division and inferred from microsatellite data (see RESULTS), predicted that populations derive from three independent refugia.

For coalescent simulations, we first estimated *N*_e _for individual populations of *G. elliotii *using values for θ calculated in MIGRATE 2.3.2 [91] under the following parameters: 10 short chains (500 trees used out of 10 000 sampled) and three long chains (5 000 trees used out of 100 000 sampled) with four adaptive heating chains (1, 3, 5 and 7). Maximum-likelihood estimates (MLE) were calculated under a stepping-stone model four times to ensure convergence upon similar values for θ. We converted θ to *N*_e _using the equation for maternally inherited mitochondrial data θ = 2 *N*_e_μ, using same mutation rate in divergence time estimate. The estimates of *N*_e _for all populations were summed to calculate Total *N*_e _and scaled the branch widths of our hypothesized population trees using the proportion of Total *N*_e _that each population comprised. The *N_e _*of the refugial population was constrained to a size proportional to the relative *N_e _*of the population sampled from the sites of the putative refugia. Thus, if samples from the site of the putative refugium had an effective population size of one-third that of the Total *N*_e_, we would constrain the population size in the simulation prior to population expansion to one-third the total *N*_e_. During coalescent simulations, both Total *N*_e _and lower and upper bounds of the 95% CI for *N_e _*were used as model parameters in order to encompass a wide range of potential *N*e values The divergence time specified for each model corresponded to the approximate age estimated using IM and IMa2 as follows: 0.1 mya for the southern and north-eastern populations (two-refugia model), 0.015 mya for northern and eastern populations (three-refugia model). In the single refugium model, we specified divergence time as 0, which assumed a panmixia population derived from a single refugium. For converting coalescent time (in generations) to absolute time, we assumed a generation length of 1 year [26].

The amount of discordance between a gene tree and population model was measured by *S*, the minimum number of sorting events required to produce the genealogy within a given model of divergence [92]. The *S *value is a measure of the number of parsimony steps in characters for a reconstructed gene tree, such that more discordance between the population model and gene tree leads to a higher *S *value. To obtain a distribution of *S *values, 10 000 gene trees were simulated, constrained within each model of population divergence under a neutral coalescent process, and the amount of discordance between the simulated genealogy and population model was determined. Overall, this produced a null distribution of 10 000 *S *values based on reconstructed genealogy for each model of population divergence. The *S *value for observed genealogy was calculated and compared to the distributions of *S *values from coalescent simulations to determine whether the observed genealogy could have been generated under a given model.

We also examined the ancestral area distributions for deeper nodes of the mitochondrial haplotype phylogeny with an event-based method in DIVA 1.1 [93]. Each individual in the phylogeographic analysis was coded to one of the ten biogeographic sectors. We used an equal likelihood for the rate of change among sectors for estimating ancestral areas because we had no information on the dispersal rate among sectors.

### Historical demography

The past population dynamics of mitochondrial lineages of *G. elliotii *were estimated using the Bayesian skyline plot method implemented in BEAST 1.4.6 [94]. This approach incorporates uncertainty in the genealogy using MCMC integration under a coalescent model, in which the timing of dates provides information about effective population sizes through time. Chains were run for 100 million generations, and the first 10% of which were discarded as 'burn-in'. The substitution model was selected according the result of Modeltest 3.7. We applied 10 grouped coalescent intervals and constant growth rate for the skyline model. The same mutation rate in divergence time estimate was used. Pilot analyses showed that the ucld.stdev parameters were close to zero, thus a strict clock model was employed. Demographic history through time was reconstructed using Tracer 1.4 [95].

The exponential growth rate (*g*) was also estimated for each mitochondrial lineage by FLUCTUATE 1.4 [96]. FLUCTUATE was initiated with a Watterson [97] estimate of theta (θ) and a random topology, performing 10 short chains, sampled every 20 genealogies for 200 steps, and two long chains, sampled every 20 genealogies for 20 000 steps. FLUCTUATE analyses were repeated five times and the mean and standard deviation of *g *calculated from the results of these separate runs. Because this genealogical method yields estimates of *g *with an upward bias [96], we corrected *g *values following the conservative approach of Lessa et al. [98] and only considered the *g *value indicative of population growth when *g *> 3 SD (*g*).

## Results

### Genetic polymorphism

A total of 4 029 bp of mitochondrial DNA was obtained, which contained 170 polymorphic sites, 125 of which were parsimony informative. These polymorphic sites defined 67 unique haplotypes, 58 of which occurred only once. Seven haplotypes were shared among individuals within the same population and two were shared between neighbouring populations. Within sampling locations, haplotype diversity values were nearly maximal, from 0.905 to 1, and nucleotide diversity ranged from 0.0028 to 0.005 (Table 1). None of the coding regions sequenced deviated significantly from neutrality (McDonald & Kreitman's test, *P *> 0.05, Table 2). The results of Fu's *F*_S _test and Fu & Li's *D *test indicated an over-abundance of singleton mutations and rare alleles for most fragments (negative *D *or *F*_S _values with *P *< 0.02, Table 2). Contradictory results between two neutrality tests and the McDonald & Kreitman's test suggest that intraspecific polymorphism of these sequences may have been mainly influenced by historical demography rather than selection.

**Table 2:** Nucleotide polymorphism and results of neutrality tests of each gene and all genes combined

	Cyt-b	COI	ND_2_	ND_6_	ATP_8_	COII	CR	Combined
Length (bp)	880	707	1003	512	217	165	545	4029
S	34	14	51	33	6	6	27	170
Nhap	32	13	34	25	6	8	27	67
Hd	0.951	0.681	0.945	0.816	0.145	0.585	0.86	0.994
π	0.0046	0.0014	0.0034	0.006	0.001	0.0013	0.0036	0.0036
Fu's *F*_S_	-18.77***	-9.86***	-25.65***	-12.84**	-5.84**	-3.96*	-23.9***	-50.38***
Fu & Li's *D*	-1.89	-3.04*	-4.48*	-3.26**	-2.57*	-2.57*	-3.54**	-4.26**
MK test	0.72	0.53	0.72	0.26	0.24	0.21		

S, number of segregating sites; Nhap, number of haplotypes; Hd, haplotype diversity; π, nucleotide diversity; Fu's *F*_S_, statistics of Fu's *F*_S _test [61] (*P < 0.02; ** *P *< 0.001; *** *P *< 0.0001); Fu & Li's *D*, statistics of Fu & Li's *D *test [62] (* *P *< 0.05; ** *P *< 0.01); MK test, probability to reject neutrality by Fisher's exact test in the McDonald & Kreitman's test [60].

For microsatellite data, there were 1 to 9 alleles per locus across all populations, and observed (*H*_O_) and expected heterozygosity (*H*_E_) ranged from 0.01 to 0.909 and 0.091 to 0.905, respectively. There was no evidence of linkage disequilibrium after adjusting the significance level for multiple comparisons (*P *< 0.001). Five locus-population combinations showed significant deviation from the Hardy-Weinberg expectation after Bonferroni correction, which involved four populations and all due to heterozygosity deficiency (Table 3).

**Table 3:** Summary of genetic variation at ten microsatellite loci for *G. elliotii *populations

Sampling location		Pocc6	Pocc8	Cum02	Cum10	Cum28	Cum32	Pat43	Lox4	Dpu15a	HeU5-A
DL	*H*_E_	0.455	0.367	0.628	0.905	0.091	0.740	0.312	0.558	0.861	0.662
	*H*_O_	0.636	0.455	0.455	0.727	0.091	0.818	0.0	0.091	0.273*	0.636
	*N_A_*	2	2	3	8	2	3	2	3	9	3
	*A*_R_	1.455	1.368	1.628	1.889	1.091	1.647	1.312	1.468	1.837	1.662
	*F*_IS_	-0.862	-0.282	-0.141	0.296	0.765	0.228	0.417	0.53	0.666	-0.076
ZD	*H*_E_	0.495	0.415	0.489	0.757	0.071	0.653	0.381	0.648	0.817	0.561
	*H*_O_	0.642	0.5	0.357	0.143*	0.071	0.857	0.214	0.429	0.286*	0.857
	*N_A_*	2	3	4	6	2	4	4	5	7	3
	*A*_R_	1.495	1.415	1.489	1.667	1.071	1.653	1.381	1.606	1.802	1.574
	*F*_IS_	-0.853	-0.295	-0.138	0.193	0.766	0.256	0.495	0.59	0.646	-0.092
WX	*H*_E_	0.495	0.363	0.67	0.593	0.703	0.813	0.264	0.703	0.747	0.791
	*H*_O_	0.714	0.429	0.857	0.571	0.286	0.286	0.286	0.286	0.286	0.286
	*N_A_*	2	2	3	5	2	4	2	3	4	4
	*A*_R_	1.495	1.363	1.67	1.593	1.533	1.758	1.264	1.703	1.747	1.727
	*F*_IS_	-0.926	-0.284	-0.04	0.284	0.687	0.025	0.565	0.55	0.667	-0.129
QL	*H*_E_		0.467	0.511	0.533		0.689	0.355	0.511	0.355	0.467
	*H*_O_		0.6	0.6	0.4		0.2	0.0	0.4	0.0	0.6
	*N_A_*		2	3	4		2	2	3	2	2
	*A*_R_		1.467	1.511	1.533		1.536	1.356	1.511	1.356	1.467
	*F*_IS_		-0.273	-0.061	0.266		0.073	0.467	0.57	0.652	-0.05
HB	*H*_E_		0.356	0.2	0.867		0.622		0.622	0.622	0.711
	*H*_O_		0.4	0.2	0.6		0.0		0.4	0.0	0.0
	*N_A_*		2	2	4		2		3	3	3
	*A*_R_		1.356	1.2	1.821		1.429		1.622	1.622	1.711
	*F*_IS_		-0.286	-0.07	0.277		0.085		0.57	0.644	-0.137
BC	*H*_E_	0.527	0.483	0.363	0.813	0.440	0.648		0.494	0.802	0.527
	*H*_O_	0.571	0.286	0.143	0.857	0	0.142		0.143	0.286	0.286
	*N_A_*	2	2	2	5	2	3		2	5	2
	*A*_R_	1.527	1.303	1.363	1.813	1.440	1.733		1.622	1.622	1.711
	*F*_IS_	-0.872	-0.288	-0.115	0.302	0.657	0.072		0.54	0.665	-0.095
YA	*H*_E_	0.4	0.12	0.592	0.858	0.325	0.875	0.233	0.742	0.575	0.6
	*H*_O_	0.5	0.125	0.75	0.875	0.375	0.125*	0.25	0.375	0.125	0.25
	*N_A_*	2	2	4	5	2	6	2	4	4	3
	*A*_R_	1.4	1.125	1.292	1.824	1.325	1.846	1.233	1.742	1.575	1.484
	*F*_IS_	-0.947	-0.287	-0.042	0.322	0.869	0.029	0.564	0.56	0.653	-0.098
MK	*H*_E_	0.582	0.476	0.669	0.783	0.632	0.706	0.632	0.743	0.804	0.632
	*H*_O_	0.786	0.714	0.857	0.429	0.214	0.786	0.143	0.357	0.286*	0.643
	*N_A_*	3	2	4	7	2	4	3	5	8	3
	*A*_R_	1.582	1.476	1.618	1.783	1.518	1.706	1.362	1.743	1.775	1.540
	*F*_IS_	-0.853	-0.205	-0.356	0.217	0.548	0.23	0.478	0.56	0.658	
CD	*H*_E_	0.363	0.473	0.648	0.78		0.67	0.274	0.714	0.802	0.626
	*H*_O_	0.429	0.571	0.714	0.285		0.714	0.143	0.143	0.714	0.857
	*N_A_*	2	3	3	5		3	3	3	5	4
	*A*_R_	1.363	1.473	1.648	1.78		1.670	1.275	1.621	1.802	1.626
	*F*_IS_	-0.872	-0.283	-0.064	0.224		0.146	0.513	0.54	0.722	-0.014
RT	*H*_E_	0.667	0.5	0.667	1	0.667	0.5	0.667		0.83	0.83
	*H*_O_	1	0.5	1	1	0.0	0.5	1		0.5	1
	*N_A_*	2	2	2	4	2	2	2		3	3
	*A*_R_	1.667	1.5	1.667	2	1.667	1.5	1.667		1.833	1.833
	*F*_IS_	0.882	-0.283	-0.052	0.276	0.698	0.125	0.578		0.671	-0.045

*H*_O_, observed heterozygosity; *H*_E_, expected heterozygosity, *N*_A_, total number of alleles, *A*_R_, allelic richness, *F*_IS_, inbreeding coefficients. Significant deviations from the Hardy-Weinberg equilibrium after adjusting the significance (alpha) level for the number of pairwise comparisons of populations and loci (*n *= 100, alpha = 0.0005). See Table 1 for abbreviations of sampling populations. Blanks indicate monomorphic loci.

### Phylogeography and population structure

**Figure 2:**
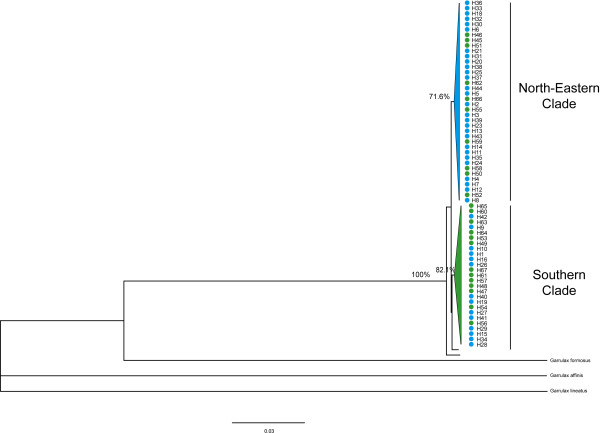
**Maximum-likelihood reconstructed phylogenetic tree of *G. elliotii *mitochondrial haplotypes**. Branch support values are shown above nodes.

A reconstructed maximum-likelihood tree of mitochondrial haplotypes suggested that *G. elliotii *was composed of two major clades with support rates of 82% and 71%, respectively (1000 replicates). The geographic distributions of the two clades appeared uneven, with the majority of first clade's haplotypes found mainly in populations in the northern and eastern subregions (north-eastern clade), whereas haplotypes of second clade were most common in southern subregion (southern clade) (Figure 2). Time to most recent common ancestor (TMRCA) for both clades fell within the late Pleistocene glacial period (Marine Isotope Stage, MIS6): 0.139 (95% HPD, 0.093-0.194) and 0.149 (95% HPD, 0.097-0.205) mya for north-eastern and southern clades, respectively.

**Figure 3:**
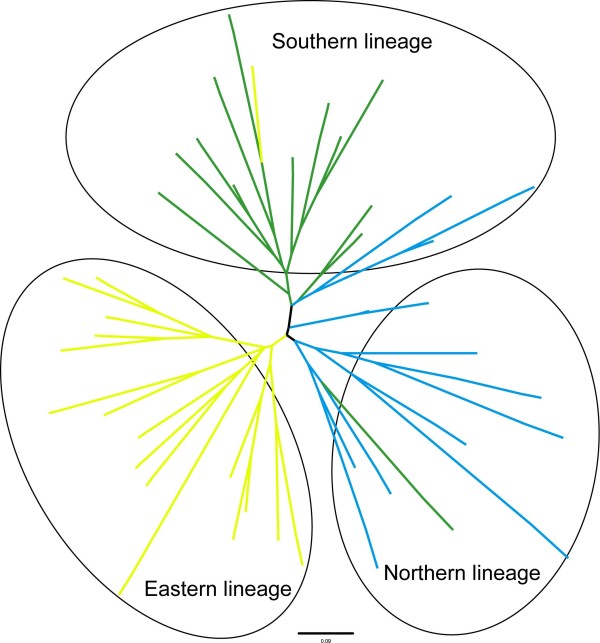
**NJ tree based on a matrix of Nei's genetic distance between *G. elliotii *individuals (calculated by GENETIX 4.05) **[62]. Colours indicate the distributions of individuals in relation to their geographical locations; green represents the southern subregion, blue the northern subregion, and yellow the eastern subregion.

**Figure 4:**
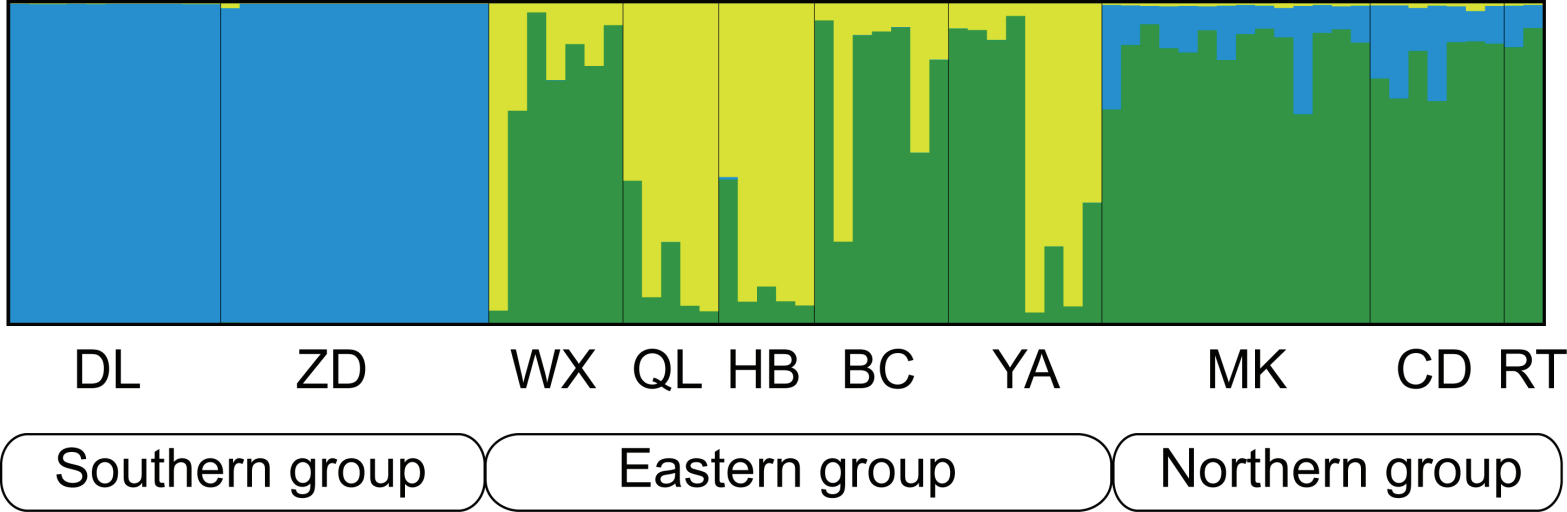
**Bar plot derived from Bayesian-based cluster analysis implemented in STRUCTURE 2.3.2. **[66]. Each column along the × axis represents one *G. elliotii *individual grouped by locations in the same order as in Table 1. The Y-axis represents the assignment probability of each individual into three clusters (*K *= 3).

Both Neighbour-Joining and STRUCTURE analyses detected similar population structures in the microsatellite data. Samples from the southern, northern and eastern subregions separated into three distinct clusters. The first cluster (southern lineage) was comprised of two populations from the southern subregion (DL and ZD), the second cluster (eastern lineage) consisted of the five eastern populations (WX, QL, HB, BC and YA), and the third cluster (northern lineage) included the three northern populations (CD, MK and RT) (Figure 3 and 4). Yet, STRUCTURE indicated that populations from the eastern subregion showed minor admixture with samples from the northern subregion (Figure 4).

To assess the genetic admixture, we carried out an exclusion method implemented in the program GENECLASS 2.0 to identify potential admixture individuals. Using the previously determined three genetic clusters and geographic sampling locations as prior population information, GENECLASS identified a considerable proportion of admixture individuals (25%), most of which were assigned to the geographically intermediate populations MK, YA and BC (method and result see Additional file 1, Table S1).

### Landscape genetics and environmental variables correlation analyses

**Figure 5:**
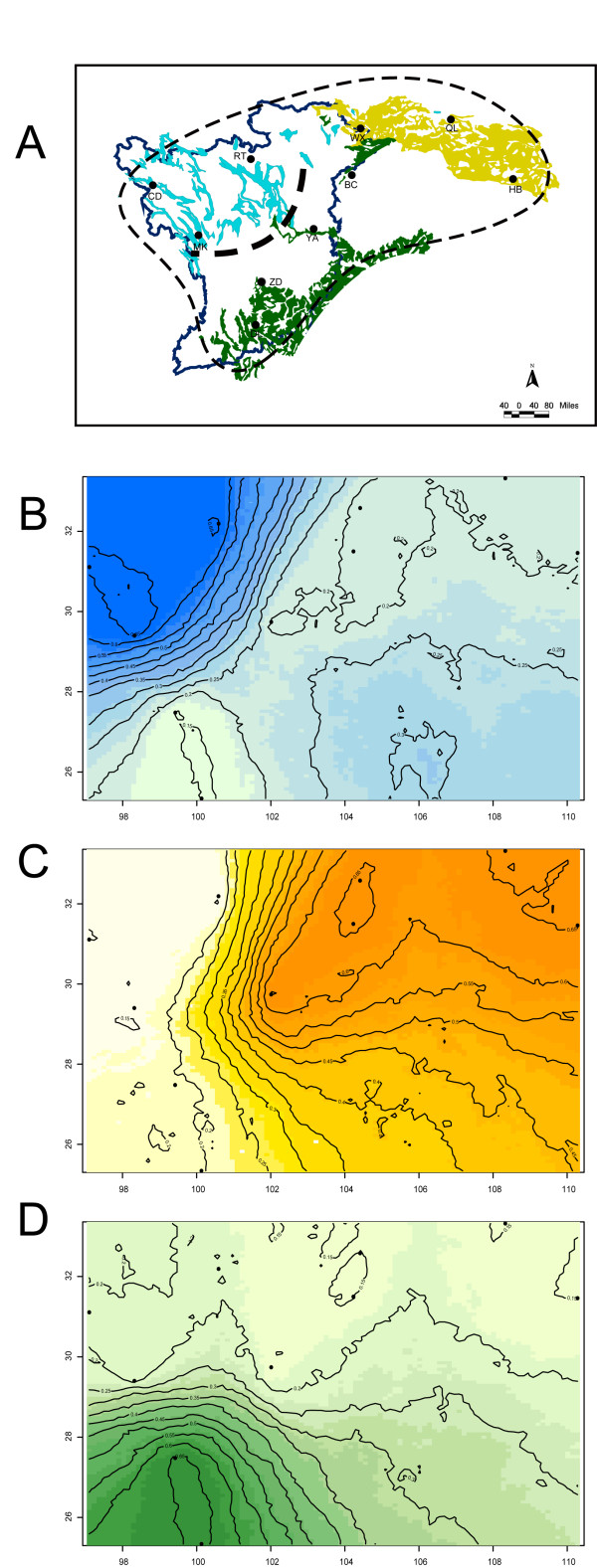
**Population structure of *G. elliotii *revealed by GENELAND 1.0.5 **[68]. (a) Map of suitable habitats of *G. elliotii*. Blue areas indicate suitable habitats that include alpine steppe, alpine and subalpine grasslands and shrublands, while green and yellow areas indicate suitable habitats that include temperate and subtropical grasslands and shrublands, respectively. (b) Northern group. (c) Eastern group. (d) Southern group. Probability of population membership increases as shading intensity decreases; solid circles show the sampling locations.

When geo-referenced microsatellite genotypes were analysed using GENELAND, we found that three Bayesian population clusters received the highest probable support (55% of estimated *K*-values from GENELAND) (Figure 5). Additionally, GENELAND revealed that a three-subregion structure was the most probable subdivision of *G. elliotii*, which verified the population structure inferred by STRUCTURE and the NJ tree. Each identified cluster was spatially contiguous and areas of steep turnover in posterior probability of population membership were presumed to reflect barriers to gene flow. Geographic information system (GIS) revealed that the three clusters are separated from adjacent clusters by areas where environmental conditions are unsuitable for *G. elliotii*. Comparing genetic divergence with spatial landscape pattern shows that gene flow barriers coincide with the spatial distribution of habitat gaps (Figure 5a).

The marginal tests revealed that three sets of environmental variables, subregion, vegetation and coordinate, were significantly correlated with genetic distance of both mitochondrial and microsatellite data. The forward selection procedure that classified variables according to the proportion of explained variation also recognized these variables sets as important variables in the multiple regression models. A combination of the three sets explained 77 to 97% of the genetic variation between locations (Table 4).

**Table 4:** Effects of environmental variables on genetic differentiation of *G. elliotii *populations based on mtDNA and microsatellite data analyses

Variable set	Marginal tests *P*	%var	Sequential tests *P*	%var
Tests for microsatellite loci and Nei's standard genetic distance				
Vegetation	0.03	66.28	0.03	66.28
Coordinate	0.001	56.66	0.355	73.62
Subregion	0.002	56.35	0.652	77.11
Elevation	0.292	13.79		
Temperature	0.444	10.39		
Rainfall	0.21	16.62		
Tests for microsatellite loci and genetic distances measured as pairwise *F*_ST_				
Coordinate	0.01	81.36	0.006	81.36
Subregion	0.005	66.2	0.075	96.66
Vegetation	0.085	70.4	0.09	96.88
Elevation	0.21	18.27		
Temperature	0.11	57.4		
Rainfall	0.1	28.4		
Tests for mtDNA and genetic distance measured as pairwise Φ_ST_				
Vegetation	0.025	77.86	0.025	77.86
Coordinate	0.028	71.61	0.020	95.05
Temperature	0.075	69.26	0.727	97.77
Subregion	0.071	46.94		
Elevation	0.160	30.84		
Rainfall	0.031	55.97		
Tests for mtDNA and genetic distance measured as uncorrected pairwise distance				
Vegetation	0.025	57.44	0.025	57.44
Temperature	0.706	31.52	0.547	73.57
Coordinate	0.588	22.39	0.532	81
Subregion	0.386	23.42		
Elevation	0.688	11.24		
Rainfall	0.450	11.64		

Marginal tests of individual variable sets as well as sequential tests of the forward selection procedure are reported. *P *indicates probability values and '%var' shows the percentage of the genetic variation explained by the particular variable. In the case of sequential tests, '%var' indicates the percentage of the genetic variation explained by the cumulative effect of variables. The top-down sequence of variables corresponds to the sequence that was indicated by the forward selection procedure.

### Divergence time estimate

As the four runs of IM and IMa2 gave similar results, we report below the estimates from the run with the highest effective sample size (ESS) for the parameter *t *(the parameter with the lowest ESS value in every run). For mitochondrial data, posterior probability for divergence time peaked at time *t *= 3.415 (90% HPD, 2.765-4.3345). When converted to a scale of years, the divergence time between north-eastern and southern lineages was estimated to be 0.109 (0.088-0.138) mya. The migration parameters estimated by IM (*m*1 and *m*2), which represented the number of migrants per mutation (*m *= m/μ), were converted to population migration rates (M = 2 Nm = θ*m*/2). The migration rate per generation was estimated to be 0.43 (0.09-0.65) from the southern to north-eastern lineage, and 0.37 (0.07-0.63) from the north-eastern to southern lineage.

For microsatellite data, the posterior probability of parameter *t *peaked at 0.975 (0.185-2.325) for the southern and north-eastern lineages, and at 0.15 (0.045-0.315) for the northern and eastern lineages. When converted to a scale of years, the divergence time between the southern and north-eastern lineages was estimated from 0.097 (0.018-0.232) to 0.01 (0.002-0.023) mya. For the northern and eastern lineages, divergence time was estimated between 0.015 (0.004-0.032) and 0.0015 (0.0005-0.003 2) mya. The migration rate per generation (2 Nm) was estimated at 0.83 (90% HPD, 0.48-1.8) from the north-eastern to southern lineage, and 1.01 (0.38-2.2) from the southern to north-eastern lineage. From the eastern to northern lineage, the migration value was estimated to be 1.34 (0.56-1.8), and 0.33 (0.02-1.14) from the northern to eastern lineage.

### Historical biogeography

All gene trees were simulated within population trees with an effective population size of *N*_e _= 2 994 038 (95% CI: 2752885 - 3235192), which equated to a MLE estimate of θ_total _of 0.0467 with lower and upper bounds of 0.0429 and 0.0505, respectively. For the observed gene tree we computed Slatkin & Maddison's *S *= 40. The discordance predicted by coalescent simulations rejected the single refugium hypothesis (mean S = 47, SD = 4, *P *< 0.05) across a range of *N*_e_. However, coalescent tests supported both the two-refugia (mean = 42, SD = 5, *P *= 0.16) and the three-refugia divergence models (mean = 40, SD = 4.5, *P *= 0.3) in three *N*_e _values.

**Figure 6:**
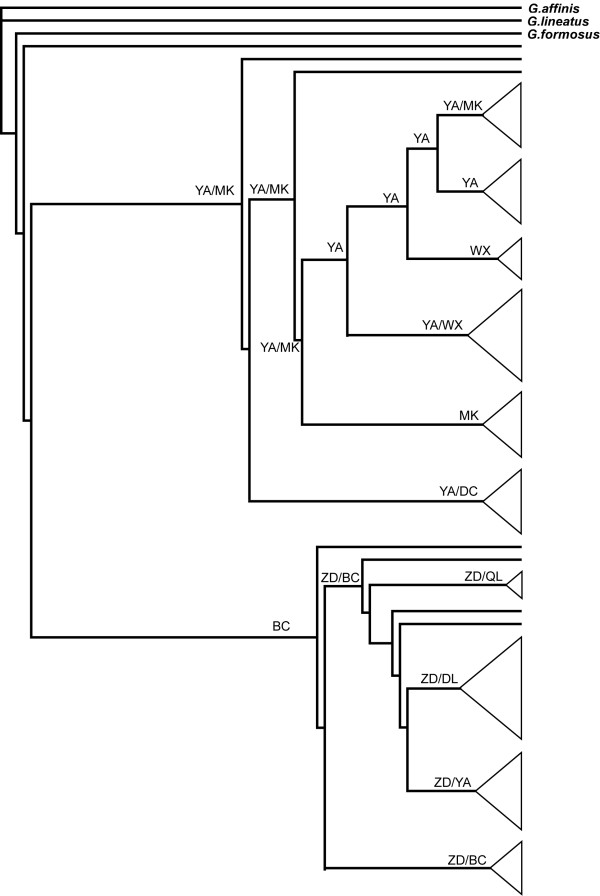
**Reconstructed ancestral areas for main internal nodes of the maximum-likelihood mitochondrial haplotype phylogeny using DIVA**.

Reconstruction of ancestral areas indicates that the north-eastern clade might have originated from YA and MK, while the southern clade probably derived from ZD and BC (Figure 6). Relatively high probabilities for multiple areas being the ancestral areas at the deeper nodes in the tree supported results from the coalescent simulation that suggest diversification occurred via multiple refugia.

### Historical demography

**Figure 7:**
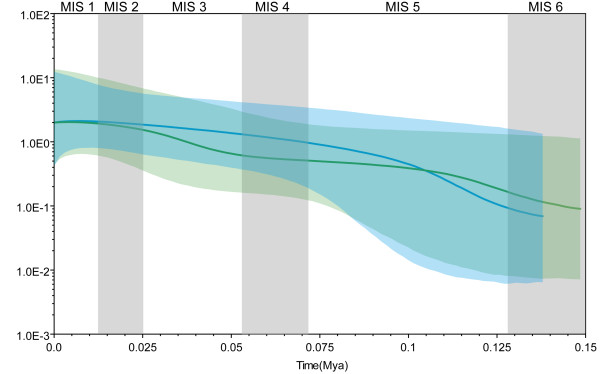
**Bayesian skyline plot representing historical demographic trends in two mitochondrial DNA lineages of *G. elliotii***. The × axis is in units of millions of years ago and is estimated based on a rate of 0.78 × 10^-8 ^substitutions per site per year. The Y axis is equal to *N_fe_*τ (the product of the effective female population size and the generation time in years (log transformed)). Estimates of means are joined by a solid line while the dashed lines delineate the 95% HPD limits. Blue represents the north-eastern lineage and green the southern lineage. The abbreviation MIS refers to marine isotope stage.

The historical population trend inferred by the Bayesian skyline plot seems a relatively good fit to the climate trend since the late Pleistocene glaciations (Figure 7). Past population dynamics of two mitochondrial lineages coherently indicate continuous population growth over the last 0.125 mya. Log transformed theta values were around 0.02 near the end of the MIS6, after which populations began to increase rapidly until they reached their current sizes (theta around 3.5). Recent population growth is also supported by maximum-likelihood estimates of positive growth rates (corrected *g*: southern clade: 1604; north-eastern clade: 3226).

## Discussion

### Pattern of distribution and biogeography

Geographic complexity and environmental heterogeneity are likely to have shaped the genetic structure among *G. elliotii *populations. Such a landscape effect is evident in the eastern Himalayan region. Although *G. elliotii *has a rather restricted geographic distribution, mitochondrial data support the separation of a southern and a north-eastern lineage with incomplete gene sorting, while microsatellites indicate a clear subdivision into three lineages, a southern, a northern and an eastern. Despite intermixing of mitochondrial haplotypes, none of the haplotypes are geographically widespread. All shared haplotypes occur within the same subregional population. In this case, the steep mountains and deep valleys of the Kangding-Muli-Baoxin Divide, recognized as a geographic barrier by Zhang et al. [19] and Li [20], appear to effectively have prevented gene flow among subregional populations. Across the entire sampling area, the correlation between phylogeographic pattern and subregion division is strong. Furthermore, our regression model reveals that the general pattern of subdivision could be attributed to geographic and environmental differentiations, e.g. subregion and vegetation.

Mountains, like sky islands, and are separated by areas of unsuitable habitats that act as barriers to gene flow [14,15]. Species restricted to sky islands commonly have high levels of interpopulation genetic divergence [8-15]. In the eastern Himalayas, *G. elliotii *is restricted to shrublands in the three subregions separated by deep valleys. The habitat-mapping model shows that these subregions or lineages are separated by areas where the environmental conditions are unsuitable for *G. elliotii*. Consistent with the expectation that these valleys prevent or restrict gene flow, each subregional population represents an evolutionary lineage. Thus, the contrast in environmental conditions at high and low elevations in the eastern Himalayas may have created a "sky island situation" for *G. elliotii*.

Although many sky island species in other regions show high levels of isolation in clusters of mountains [7-14], our data indicate that diversification of *G. elliotii *has occurred on a broader geographic scale, eco-subregions. *G. elliotii *is an alpine bird found between 2 000 and 4 000 m.a.s.l. [24-26]. This broad altitudinal range implies large ecological plasticity. Considering that most previously studied sky island species belong to less mobile groups such as beetles and plants, this difference in scale probably reflects the fact that historical processes of isolation are more easily reconstructed in species with less dispersal capability than in highly mobile species [1-4].

While our study may demonstrate newly discovered sky island diversification in a previously unstudied region, sky islands also have the extra connotation that mountain ranges are equivalent ecologically and they share the same biogeographic history. Since three eco-subregions in the eastern Himalayas present different ecological systems, our result should be considered cautiously. Whether the eastern Himalayan region is a sky island situation ultimately depends on the ecology of the organisms under study. Further research on more organisms inhabiting this region, especial the same eco-climatic subregions is required to clarify this.

### Effects of Pleistocene glaciations on population divergence

The Pleistocene glacial cycles have been considered to induce environmental shifts in the eastern Himalayas resulting in the isolation of *G. elliotii *populations on different subregions. Congruent with this, our divergence time estimates indicate that lineage diversification occurred during the late Pleistocene interglacial period (MIS5). Climatic fluctuations during the late Pleistocene resulted in several glacial-interglacial cycles in the eastern Himalayas [27,28,99-101]. Glaciations were restricted to relatively high altitudes and did not affect the lower slopes or valleys [27]. Palaeoclimatic and palynological studies from this region reveal a vegetational shift over the Pleistocene glacial cycles [102,103]; during the glacial periods, cool-temperate vegetations, such as shrublands, expanded to lower elevations, and contracted to high elevations during warmer and wetter interglacial periods [104,105]. Because alpine birds are often strongly associated with their preferred habitat, range expansion and contraction of shrublands probably mirrored that of *G. elliotii*. It is plausible therefore that the glacial and interglacial cycles would allow *G. elliotii *to disperse among subregions during glacial periods, but isolate population in different subregions during the interglacial periods.

Given the potential for repeated contacts of populations from subregions during glacial expansions, it is possible that subregional populations merged into a common gene pool. However, our analyses of diversification pattern in *G. elliotii *appear to reflect historical differentiation in multiple refugia. Coalescent simulations and reconstruction ancestral areas of the mtDNA phylogeny suggested a divergence model involving the multiple refugia, while microsatellite data identify a distinct genetic structure concordant with the geographically separated southern, northern and eastern subregions. These results suggested that subregional populations of *G. elliotii *were isolated into separated refugia during down-slope expansions, although we could not distinguish between the two-refugia and three-refugia model. Furthermore, while coalescent simulation assumes that the discordance between the reconstructed gene tree and multi-refugia population trees reflects the retention of ancestral variation, the discordance could also result from migration among subregional populations (see below).

### Genetic admixture among intraspecific lineages

In contrast to the deep phylogeographic partitions found in other sky island species [8-14], we only found a shallow phylogeographic division in *G. elliotii*. This is consistent with genetic admixture among lineages occurring during glacial periods. The two partially sympatric mitochondrial lineages show shared ancestry, and microsatellite data indicate gene flow among the three subregional populations. Furthermore, GENECLASS identified a considerable proportion of admixed individuals, most of which were assigned to the geographically intermediate populations MK, YA and BC. Interestingly, despite the relatively high degree of genetic admixture, these locations were also identified as the potential refugia that most likely defined the boundary of each genetic lineage.

Based on these observations, it is probable that isolated populations of *G. elliotii *periodically expanded to lower elevation where they mixed. Thus, the three intraspecific lineages likely established a contact zone along their boundaries. Because these lineages are geographically relatively close and are likely to have experienced repeated glacial cycles, this has resulted in multiple episodes of genetic admixture. Over time, this could have defined a centre of gene flow, such as populations YA, MK and BC. This process might cause the mixing of genetic lineages thereby obscuring the pattern of genetic variation [42].

Nevertheless, the rather large degree of genetic separation among lineages indicates that these have been isolated from each other for a relatively long period. Although the southern and north-eastern mitochondrial lineages are intermixed, no haplotypes are shared between them. This lack of sharing suggests that the two lineages are at an intermediate stage of divergence along the continuum from complete panmixia to paraphyly and ultimately to reciprocal monophyly [106,107]. In contrast, differences in microsatellite allele frequencies among the three lineages are more distinct. The discrepancy between these two markers may indicate that admixture among these lineages is relatively ancient, and hence, that the mixing signature is relatively weak.

### Historical demography

Climatic changes that resulted in population expansion and contraction are also expected to lead to demographic changes over time between lineages confined to different subregions. Congruent with this, the Bayesian skyline plot revealed an increasing population size in each genealogical lineage since 0.125 mya, i.e. during the warmer interglacial MIS5 period. During the Quaternary, the eastern Himalayas were uplifted to 4 000 - 4 500 m.a.s.l. Climatic conditions responsible for high rainfall moved away from the centre of mountains and mountain glaciers shrank [27,99]. The maximum extent of glacial development in this region occurred during MIS6 and MIS4, with ice being restricted during the global Late Glacial Maximum, LGM (MIS2) [27-29]. Palynological research has indicated that a series of prolonged, mild interglacials supported vegetation similar to the present-day flora of East Asia during MIS5 (0.11 to 0.071 mya) [42,108,109]. It is likely that Pleistocene climatic stability might have allowed the persistence of vegetation similar to that observed today in the eastern Himalayas, especially at moderate or low altitudes [109]. Therefore, our results, combined with the palaeoclimatic and palynological data, suggest that *G. elliotii *experienced population growth during the warmer MIS5 period.

Contrary to the expectation that profound ecological upheavals during cooler periods would have reduced the population size of *G. elliotii*, the Bayesian skyline plot revealed a stable population during the cooler MIS4 and MIS2 periods. Maintenance of a rather stable population size can probably be attributed to the frequency and location of glaciations in this region. Unlike high latitude regions of Europe and North America that were covered by heavy ice during much of Pleistocene, glaciations in the eastern Himalayas were restricted to relatively high altitudes and did not affect the lower slopes or valleys [26]. It is likely that this relatively milder climate was less stressful for cold-tolerant alpine birds than the extremes experienced by both European and North American birds. Although temperatures might have been lower elsewhere during the MIS4 and MIS2, it has been inferred that the climate of the eastern Himalayas, particularly at low altitudes, was warmer [42,107-109]. Therefore, the resultant stable niches might have provided suitable habitats for *G. elliotii *even during periods of climatic change, making it possible for this species to maintain a stable population size during the cooler MIS4 and MIS2.

## Conclusion

The genetic structure observed in *G. elliotii *indicates that lineages have been isolated on individual subregions since the late Pleistocene interglacial period (MIS 5). Despite being isolated in multiple refugia during glacial advance, gene flow periodically occurred when populations expanded their ranges to lower altitudes. The resultant genetic admixture might have caused the mixing of genetic lineages thereby obscuring the pattern of genetic variation. The Bayesian skyline plot shows a gradual increase in population size in each mitochondrial lineage since the late Pleistocene interglacial period (MIS 5). Our results provide new evidence that climatic changes during the Pleistocene glaciations had profound effects on lineage diversification and demography in a bird species in a previously unstudied montane region - the eastern Himalayas.

## Authors' contributions

YHQ, XL and FML conceived and designed the study. RYZ performed lab work. XL, GS and FSZ collected most of the samples. All authors read, made significant comments and approved the final manuscript.

## Supplementary Material

Additional file 1 **Table S1**. The geographic origins and GENECLASS assigned groups for the admixed individuals [111]; 1 represents the southern group; 2, the eastern group; 3, the northern group. The likelihood of an individual's genotype belonging to the population where the individual has been sampled was estimated using the frequency-based method [112]. The probability that an individual was not from the local population was computed using a gamete-based Monte Carlo resampling method with 1 000 simulated individuals and a threshold of 0.01 [113]. Click here for file

## References

[B1] Avise JC Phylogeography: The History and Formation of Species 2000 Cambridge, Massachusetts: Harvard University Press

[B2] Hewitt GM Some genetic consequences of ice ages and their role in divergence and speciation Biological Journal of the Linnean Society 1996 58 247 276

[B3] Hewitt GM The genetic legacy of the Quaternary ice ages Nature 2000 405 907 913 10.1038/35016000 10879524

[B4] Hewitt GM Genetic consequences of climatic oscillations in the Quaternary. Philosophical Transactions of the Royal Society of London Series B Biological Sciences 2004 359 183 195 10.1098/rstb.2003.1388 PMC1693318 15101575

[B5] Wiens JJ Speciation and ecology revisited: a phylogenetic niche conservatism and the origin of species Evolution 2004 58 193 197 15058732 10.1111/j.0014-3820.2004.tb01586.x

[B6] Maddison WP Gene trees in species trees Systematic Biology 1997 46 523 536 10.1093/sysbio/46.3.523

[B7] Knowles LL Tests of Pleistocene speciation in montane grasshoppers (genus *Melanoplus*) from the sky islands of western North America Evolution 2000 54 1337 1348 11005300 10.1111/j.0014-3820.2000.tb00566.x

[B8] Knowles LL Did the Pleistocene glaciations promote divergence? Tests of explicit refugial models in montane grasshoppers Molecular Ecology 2001 10 691 701 11298980 10.1046/j.1365-294x.2001.01206.x

[B9] Maddison WP McMahon M Divergence and reticulation among montane populations of a jumping spider (*Habronattus pugillis *Griswold) Systematic Biology 2000 49 400 421 10.1080/10635159950127312 12116419

[B10] Masta SE Phylogeography of the jumping spider *Habronattus pugillis *(Araneae: Salticidae): recent vicariance of sky island populations? Evolution 2000 54 1699 1711 11108597 10.1111/j.0014-3820.2000.tb00714.x

[B11] Smith CI Farrell BD Phylogeography of the longhorn cactus beetle *Moneilema appressum *LeConte (Coleoptera: Cerambycidae): was the differentiation of the Madrean sky islands driven by Pleistocene climate changes? Molecular Ecology 2005 14 3049 3065 10.1111/j.1365-294X.2005.02647.x 16101773

[B12] Carstens BC Knowles LL Shifting distributions and speciation: Species divergence during rapid climate change Molecular Ecology 2007 16 619 627 17257117 10.1111/j.1365-294X.2006.03167.x

[B13] Shepard DB Burbrink FT Lineage diversification and historical demography of a sky island salamander, *Plethodon ouachitae*, from the Interior Highlands Molecular Ecology 2008 17 5315 5335 10.1111/j.1365-294X.2008.03998.x 19121000

[B14] Shepard DB Burbrink FT Phylogeographic and demographic effects of Pleistocene climatic fluctuations in a montane salamander, *Plethodon fourchensis* Molecular Ecology 2009 18 2243 2262 10.1111/j.1365-294X.2009.04164.x 19389165

[B15] Patriat P Achache J India-Eurasia collision chronology has implications for crustal shortening and driving mechanisms of plates Nature 1984 311 615 621 10.1038/311615a0

[B16] Wang CS Ding XL The new research in progress of Tibet Plateau uplift Advances in Earth Science 1998 13 526 532

[B17] Tao JR The Tertiary vegetation and flora and floristic regions in China Acta Phytotaxonomica Sinica 1992 30 25 43

[B18] Tao JR The Evolution of the Late Cretaceous-Cenozoic Floras in China 2000 Beijing: Science Press

[B19] Zhang DC Boufford DE Ree RH Sun H The 29°N latitudinal line: an important division in the Hengduan mountains, a biodiversity hotspot in southwest China Nordic Journal of Botany 2009 27 405 412 10.1111/j.1756-1051.2008.00235.x

[B20] Li BY On the boundaries of the Hengduan Mountains Mountain Research 1989 7 15 20

[B21] Huang XL Qiao GX Lei FM Use of parsimony analysis to identify areas of endemism of Chinese birds: implications for conservation and biogeography International Journal of Molecular Sciences 2010 11 2097 2108 10.3390/ijms11052097 20559504 PMC2885096

[B22] Sun H Evolution of Arctic-Tertiary flora in Himalayan-Hengduan Mountains Acta Botanica Yunnanica 2002 24 671 688

[B23] Li JJ Wang XM Ma HZ Geomorphological and environmental evolution in the upper reaches of Huanghe River during the late Cenozoic Science in China, Series D 1996 39 80

[B24] Tang CZ Birds of the Hengduan Mountains Region 1996 Beijing: Sciences Press (In Chinese)

[B25] Cheng TH Long ZY Zheng BL Fauna Sinica (Aves, Vol 11 Passeriformes, Muscicapidae II Timaliinae) 1987 Beijing: Science Press (In Chinese)

[B26] Cheng TH On the evolution of *Garrulax *(Timaliinae), with comparative studies of the species found at the center and those in the periphery of the distributional range of the genus Acta Zoologica Sinica 1982 28 205 209 (In Chinese)

[B27] Zhou S Wang X Wang J Xu L A preliminary study on timing of the oldest Pleistocene glaciation in Qinghai-Tibetan plateau Quaternary International 2006 154-155 44 51

[B28] Benn DI Owen LA The role of the Indian summer monsoon and the mid-latitude westerlies in Himalayan glaciation: review and speculative discussion Journal of Geological Society 1998 155 353 363 10.1144/gsjgs.155.2.0353

[B29] Zhang W Cui Z Li Y Review of the timing and extent of glaciers during the last glacial cycle in the bordering mountains of Tibet and in East Asia Quaternary International 2006 154-155 32 43

[B30] Zhang RZ Zheng D Yang QY Liu YH Physical geography of Hengduan Mountains 1997 Beijing: Science Press

[B31] Lei FM Qu YH Lu JL Liu Y Yin ZH Conservation on diversity and patterns of endemic birds in China Biodiversity and Conservation 2003 12 239 254 10.1023/A:1021928801558

[B32] Lei FM Qu YH Tan QQ An SC Priorities for the conservation of avian biodiversity in China based on the distribution patterns of endemic bird genera Biodiversity and Conservation 2003 12 2487 2501 10.1023/A:1025886718222

[B33] Nie SR Natural Geography of Shaanxi 1981 Xian: Shaanxi People Press

[B34] Bachman S Baker WJ Brummitt N Dransfield J Moat J Elevational gradients, area and tropical island diversity: an example from the palms of New Guinea Ecography 2004 27 299 310 10.1111/j.0906-7590.2004.03759.x

[B35] Sanders NJ Elevational gradients in ant species richness: area, geometry, and Rapoport's rule Ecography 2002 25 25 32 10.1034/j.1600-0587.2002.250104.x

[B36] Fu C Wu JH Wang XY Lei GC Chen JK Patterns of diversity, altitudinal range and body size among freshwater fishes in the Yangtze River basin, China Global Ecology and Biogeography 2004 13 543 552 10.1111/j.1466-822X.2004.00122.x

[B37] Fu J Weadick CJ Bi K A phylogeny of the high elevation Tibetan megophryid frogs and evidence for the multiple origins of reversed sexual size dimorphism Journal of Zoology 2007 273 315 325 10.1111/j.1469-7998.2007.00330.x

[B38] Rickart EA Elevational diversity gradients, biogeography and the structure of montane mammal communities in the intermountain region of North America Global Ecology and Biogeography 2001 10 77 100 10.1046/j.1466-822x.2001.00223.x

[B39] Edwards SV Wilson AC Phylogenetically informative length polymorphism and sequence variability in mitochondrial DNA of Australian songbirds (*Pomatostomus*) Genetics 1990 126 695 711 1979038 10.1093/genetics/126.3.695 PMC1204224

[B40] Hebert PDN Stoeckle MY Zemlak TS Francis CM Identification of birds through DNA barcodes PLoS Biology 2004 2 1657 1663 10.1371/journal.pbio.0020312 PMC518999 15455034

[B41] Kocher TD Thomas WK Meyer A Edwards SV Paabo S Villablanca FX Wilson AC Dynamics of mitochondrial DNA evolution in animals: amplification and sequencing with conserved primers Proceedings of the National Academy of Sciences of the United States of America 1989 86 6196 6200 10.1073/pnas.86.16.6196 2762322 PMC297804

[B42] Li SH Yeung CKL Feinstein J Han LX Le MH Wang CX Ding P Sailing through the Late Pleistocene: unusual historical demography of an East Asian endemic, the Chinese Hwamei (*Leucodioptron canorum canorum*), during the last glacial Period Molecular Ecology 2009 18 622 633 10.1111/j.1365-294X.2008.04028.x 19215583

[B43] Sorenson MD Ast JC Dimcheff DE Yuri T Mindell DP Primers for a PCR-based approach to mitochondrial genome sequencing in birds and other vertebrates Molecular Phylogenetics and Evolution 1999 12 105 114 10.1006/mpev.1998.0602 10381314

[B44] Tarr CL Primers for amplification and determination of mitochondrial control-region sequences in oscine passerines Molecular Ecology 1995 4 527 529 10.1111/j.1365-294X.1995.tb00251.x 8574451

[B45] Qu YH Ericson PGP Lei FM Li SH Post-glacial colonization of the Qinghai-Tibetan plateau inferred from matrilineal genetic structure of the endemic red-necked snow finch, *Pyrgilauda ruficollis* Molecular Ecology 2005 14 1767 1781 10.1111/j.1365-294X.2005.02528.x 15836648

[B46] Primmer CR Moller AP Ellegren H Resolving genetic relationships with microsatellite markers: a parentage testing system for the swallow, *Hirundo rustica* Molecular Ecology 1995 4 493 498 10.1111/j.1365-294X.1995.tb00243.x 8574445

[B47] Piertney SB Marquiss M Summers R Characterization of tetranucleotide microsatellite markers in the Scottish crossbill (*Loxia scotica*) Molecular Ecology 1998 7 1261 1263 9734087

[B48] Gibbs HL Tabak LM Hobson K Characterization of microsatellite DNA loci for a neotropical migrant songbird, the Swainson's thrush (*Catharus ustulatus*) Molecular Ecology 1999 8 1551 1552 10.1046/j.1365-294x.1999.00673.x 10564463

[B49] Otter K Ratcliffe L Michaud D Boag PT Do female blackcapped chickadees prefer high-ranking males as extra-pair partners? Behavioral Ecology and Sociobiology 1998 43 25 36 10.1007/s002650050463

[B50] Bensch S Price T Kohn J Isolation and characterization of microsatellite loci in a *Phylloscopus *warbler Molecular Ecology 1997 6 91 92 10.1046/j.1365-294X.1997.00150.x 9004521

[B51] Dawson RJG Gibbs HL Hobson KA Yezerinac SM Isolation of microsatellite DNA markers from a passerine bird, *Dendroica petechia *(the yellow warbler), and their uses in population studies Heredity 1997 79 506 514 10.1038/hdy.1997.190 9369012

[B52] Rozas J Sánchez-De I Barrio JC Messeguer X Rozas R DnaSP, DNA polymorphism analyses by the coalescent and other methods Bioinformatics 2009 19 2496 2497 10.1093/bioinformatics/btg359 14668244

[B53] McDonald JH Kreitman M Adaptive protein evolution at the Adh locus in *Drosophila* Nature 1991 351 652 654 10.1038/351652a0 1904993

[B54] Fu YX Statistical tests of neutrality of mutations against population growth, hitchhiking and background selection Genetics 1997 147 915 925 9335623 10.1093/genetics/147.2.915 PMC1208208

[B55] Fu YX Li WH Statistical tests of neutrality of mutations Genetics 1993 133 693 709 8454210 10.1093/genetics/133.3.693 PMC1205353

[B56] Excoffier L Laval G Schneider S Arlequin ver. 3.0: An integrated software package for population genetics data analysis Evolutionary Bioinformatics Online 2005 1 47 50 19325852 PMC2658868

[B57] Goudet J FSTAT version 1.2: a computer program to calculate *F*-statistics Journal of Heredity 1995 86 485 486

[B58] Guindon S Gascuel O A simple, fast, and accurate algorithm to estimate large phylogenies by maximum likelihood Systematic Biology 2003 52 696 704 10.1080/10635150390235520 14530136

[B59] Akaike H Petrov BN, Csaki F Information theory as an extension of the maximum likelihood principle Second International Symposium on Information Theory 1973 Budapest: Akademiai Kiado 267 281

[B60] Posada D Crandall KA Modeltest: testing the model of DNA substitution Bioinformatics 1998 14 817 818 10.1093/bioinformatics/14.9.817 9918953

[B61] Luo X Qu YH Han LX Li SH Lei FM A Phylogenetic analysis of laughing thrushes (Timaliidae: *Garrulax*) and allies based on mitochondrial and nuclear DNA sequences Zoologica Scripta 2009 38 9 22 10.1111/j.1463-6409.2008.00355.x

[B62] Belkhir K Borsa P Chikhi L Raufaste N Bonhomme F GENETIX 4.05, Windows Software for Population Genetics 2004 Montpellier: Laboratoire Génome, Populations, Université de Montpellier II

[B63] Saitou N Nei M The neighbor-joining method - A new method for reconstructing phylogenetic trees Molecular Biology and Evolution 1987 4 406 425 3447015 10.1093/oxfordjournals.molbev.a040454

[B64] Felsenstein J PHYLIP (Phylogeny Inference Package), version 3.5. Distributed by the Author 1993 Seattle: Department of Genetics, University of Washington

[B65] Page RDM TreeView: an application to display phylogenetic trees on personal computer Computer Applications in the Biosciences 1996 12 357 358 8902363 10.1093/bioinformatics/12.4.357

[B66] Pritchard JK Stephens M Donnelly P Inference of population structure using multilocus genotype data Genetics 2000 155 945 959 10835412 10.1093/genetics/155.2.945 PMC1461096

[B67] Evanno G Regnaut S Goudet J Detecting the number of clusters of individuals using the software structure: a simulation study Molecular Ecology 2005 14 2611 2620 10.1111/j.1365-294X.2005.02553.x 15969739

[B68] Guillot G Mortimer F Estoup A Geneland: a computer package for landscape genetics Molecular Ecology Notes 2005 5 712 715 10.1111/j.1471-8286.2005.01031.x

[B69] Ihaka R Gentleman R R: a language for data analysis and graphics Journal of Computational and Graphical Statistics 1996 5 299 314 10.2307/1390807

[B70] Coulon A Guillot G Cosson JF Angibault JMA Aulagnier S Cargnelutti B Galan M Hewison AJM Genetic structure is influenced by landscape features: empirical evidence from a roe deer population Molecular Ecology 2006 15 1669 1679 10.1111/j.1365-294X.2006.02861.x 16629819

[B71] Legendre P Anderson MJ Distance-based redundancy analysis: testing multispecies responses in multifactorial ecological experiments Ecological Monographs 1999 69 1 24 10.1890/0012-9615(1999)069[0001:DBRATM]2.0.CO;2

[B72] McArdle BH Anderson MJ Fitting multivariate models to community data: a comment on distance-based redundancy analysis Ecology 2001 82 290 297 10.1890/0012-9658(2001)082[0290:FMMTCD]2.0.CO;2

[B73] Anderson MJ DISTLM Version 5: a FORTRAN Computer Program to Calculate a Distance-Based Multivariate Analysis for a Linear Model 2004 New Zealand: Department of Statistics, University of Auckland Available at: http://www.stat.auckland.ac.nz/~mja/Programs.htm

[B74] Anderson MJ DISTLM Forward: a FORTRAN Computer Program to Calculate a Distance-Based Multivariate Analysis for a Linear Model Using Forward Selection 2003 New Zealand: Department of Statistics, University of Auckland Available at: http://www.stat.auckland.ac.nz/~mja/Programs.htm

[B75] Beerli P Effect of unsampled populations on the estimation of population sizes and migration rates between sampled populations Molecular Ecology 2004 13 827 836 10.1111/j.1365-294X.2004.02101.x 15012758

[B76] Nielsen R Wakeley J Distinguishing migration fromisolation: a Markov chain Monte Carlo approach Genetics 2001 158 885 896 11404349 10.1093/genetics/158.2.885 PMC1461674

[B77] Whitlock MC McCauley DE Indirect measures of gene flow and migration: *F*_ST _not equal to 1/(4*N*_m _+ 1) Heredity 1999 82 117 125 10.1038/sj.hdy.6884960 10098262

[B78] Hey J Nielsen R Multilocus methods for estimating population sizes, migration rates and divergence time, with applications to the divergence of *Drosophila pseudoobscura *and *D. persimilis* Genetics 2004 167 747 760 10.1534/genetics.103.024182 15238526 PMC1470901

[B79] Hey J On the number of New World founders: a population genetics portrait of the peopling of the America PLoS Biology 2005 3 965 975 10.1371/journal.pbio.0030193 PMC1131883 15898833

[B80] Won YJ Hey J Divergence population genetics of chimpanzees Molecular Biology and Evolution 2005 22 297 307 15483319 10.1093/molbev/msi017

[B81] Nielsen R Wakeley J Distinguishing migration from isolation: a Markov chain Monte Carlo approach Genetics 2001 158 885 896 11404349 10.1093/genetics/158.2.885 PMC1461674

[B82] Klicka J Zink RM The importance of recent ice ages in speciation: a failed paradigm Science 1997 277 1666 1669 10.1126/science.277.5332.1666

[B83] Weir JT Schluter D Calibrating the avian molecular clock Molecular Ecology 2008 17 2321 2328 10.1111/j.1365-294X.2008.03742.x 18422932

[B84] Fleischer RC Boarman WI Gonzalez EG Godinez A Omland KE Young S Helgen L Syed G Mcintosh CE As the rave flies: using genetic data to infer the history of invasive common raven (*Corvus corax*) populations in the Mojave Desert Molecular Ecology 2008 17 464 474 10.1111/j.1365-294X.2007.03532.x 17908216

[B85] Akst EP Boersma PD Fleischer RC A comparison of genetic diversity between the Galapagos Penguin and the Magellanic Penguin Conservation Genetics 2002 3 375 383 10.1023/A:1020555303124

[B86] Gibbs HL Brooke MDL Davies NB Analysis of genetic differentiation of host races of the common cuckoo *cuculus canorus *using Mitochondrial and Microsatellite DNA variation Proceedings of the Royal Society B: Biological Sciences 1996 263 89 96 10.1098/rspb.1996.0015 8587899

[B87] Hillel J Groenen MAM Tixier-Boichard M Korol AB David L Kirzhner M Burke T Barre-Dirie AB Crooijmans RPMA Elo K Feldman MW Freidlin PJ Maki-Tanila AM Oortwijn M Thomson P Vignal A Wimmers K Weigend S Biodiversity of 52 chicken populations assessed by microsatellite typing of DNA pools Genetic Selection Evolution 2003 35 533 557 10.1186/1297-9686-35-6-533 PMC2697980 12939204

[B88] Mundy NI Winchell CS Burr T Woodruff DS Microsatellite variation and microevolution in the critically endangered San Clemente island loggerhead shrike (*Lanius ludovicianus mearnsi*) Proceedings of the Royal Society B: Biological Sciences 1997 264 869 875 10.1098/rspb.1997.0121

[B89] Primmer CR Saino N Moller AP Ellegren H Unravelling the processes of microsatellite evolution through analysis of Germ line mutations in Barn swallows *Hirundo rustica* Molecular Biology and Evolution 1998 15 1047 1054

[B90] Maddison WP Maddison DR Mesquite: a modular system for evolutionary analysis 2008 Http://mesquiteproject.org Version 2.5

[B91] Beerli P Felsenstein J Maximum likelihood estimation of a migration matrix and effective population sizes in n subpopulations by using a coalescent approach Proceedings of the National Academy of Sciences of the USA 2001 98 4563 4568 http://popgen.sc.fsu.edu/Migrate/Migrate-n.html 10.1073/pnas.081068098 11287657 PMC31874

[B92] Slatkin M Maddison WP A cladistic measure of gene flow inferred from the phylogenies of alleles Genetics 1989 123 603 613 2599370 10.1093/genetics/123.3.603 PMC1203833

[B93] Ronquist F DIVA version1.1 1996 Computer program and manual available by anonymous FTP from Uppsala University

[B94] Drummond AJ Rambaut A Beast V1.4 Bayesian evolutionary analysis sampling trees 2006 Available from: http://beast.bio.ed.ac.uk/Main_Page 10.1186/1471-2148-7-214 PMC2247476 17996036

[B95] Rambaut A Drummond AJ Tracer V1.3 2005 available from: http://beast.bio.ed.ac.uk/tracer

[B96] Kuhner M Yamato J Felsenstein J Maximum likelihood estimation of population growth rates based on the coalescent Genetics 1998 149 429 434 9584114 10.1093/genetics/149.1.429 PMC1460154

[B97] Watterson GA On the number of segregating sites in genetic models without recombination Theoretical Population Biology 1975 7 256 276 10.1016/0040-5809(75)90020-9 1145509

[B98] Lessa EP Cook JA Patton JL Genetic footprints of demographic expansion in North America, but not Amazonia, during the Late Quaternary Proceeding of the National Academy of Sciences, USA 2003 100 10331 10334 10.1073/pnas.1730921100 PMC193561 12913123

[B99] Zheng B Xu Q Shen Y The relationship between climate change and Quaternary glacial cycles on the Qinghai-Tibetan plateau: review and speculation Quaternary International 2002 97-98 93 101

[B100] Zhang W Cui Z Li Y Review of the timing and extent of glaciers during the last glacial cycle in the bordering mountains of Tibet and in East Asia Quaternary International 2006 154-155 32 43

[B101] Liu J Yu G Chen X Paleoclimatic simulation of 21 ka for the Qinghai-Tibetan plateau and eastern Asia Climatic Dynamics 2002 19 575 583 10.1007/s00382-002-0248-6

[B102] Kou XY Ferguson DK Xu JX Wang YF Li CS The reconstruction of paleovegetation and paleoclimate in the Late Pliocene of West Yunnan, China Climatic Change 2006 77 431 448 10.1007/s10584-005-9039-5

[B103] Yu G Gui F Shi Y Zheng Y Late marine isotope stage 3 palaeoclimate for East Asia: a data-model comparison Palaeogeography, Palaeoclimatology, Palaeoecology 2007 250 167 183 10.1016/j.palaeo.2007.03.010

[B104] Wang YJ Cheng H Edwards RL A high-resolution absolute-dated Late Pleistocene monsoon record from hulu cave, China Science 2001 294 2345 2348 10.1126/science.1064618 11743199

[B105] Neigel JE Avise JC Nevo E, Karlin S Phylogenetic relationships of mitochondrial DNA under various demographic models of speciation Evolutionary Processes and Theory 1986 New York: Academic Press 513 534

[B106] Zink RM Drovetski SV Questiau S Fadeev IV Recent evolutionary history of the bluethroat (*Luscinia svecica*) across Eurasia Molecular Ecology 2003 12 3069 3075 10.1046/j.1365-294X.2003.01981.x 14629386

[B107] Kelly MJ Edwards RL Cheng H Yuan D Cai Y Zhang M Lin Y An Z High resolution characterization of the Asian Monsoon between 146 000 and 99 000 years B.P. from Dongge Cave, China and global correlation of events surrounding Termination II Palaeogeography, Palaeoclimatology, and Palaeoecology 2006 236 20 38 10.1016/j.palaeo.2005.11.042

[B108] Yuan DX Cheng H Edwards RL Timing, duration, and transitions of the last interglacial Asian Monsoon Science 2004 304 575 578 10.1126/science.1091220 15105497

[B109] Liu C Xie Z Liu S Kang E Glacial water resources and their change in the arid northwestern China Glacier-Snow water resources and mountain runoff in the arid area of northwest China 2002 Beijing: Science Press 14 51

[B110] MacKinnon J Phillipps K He F A field guide of the birds of China 2000 Oxford University Press

[B111] Piry S Alapetite A Cornuet JM Paetkau D Baudouin L Estoup A GENECLASS2: a software for genetic assignment and firstgeneration migrant detection Journal of Heredity 2004 95 536 539 10.1093/jhered/esh074 15475402

[B112] Paetkau D Calvert W Stirling I Strobeck C Microsatellite analysis of population structure in Canadian polar bears Molecular Ecology 1995 4 347 354 10.1111/j.1365-294X.1995.tb00227.x 7663752

[B113] Paetkau D Slade R Burden M Estoup A Direct, real-time estimation of migration rate using assignment methods: a simulation-based exploration of accuracy and power Molecular Ecology 2004 13 55 65 10.1046/j.1365-294X.2004.02008.x 14653788

