# Supplementary material for: Lineage diversification and historical demography of a montane bird Garrulax elliotii - implications for the Pleistocene evolutionary history of the eastern Himalayas
Source: BMC Evol Biol. 2011 Jun 21;11:174. doi: 10.1186/1471-2148-11-174 (PMC3150279; doi:10.1186/1471-2148-11-174)
Supplement: Additional file 1 — Table S1. The geographic origins and GENECLASS assigned groups for the admixed individuals [111]; 1 represents the southern group; 2, the eastern group; 3, the northern group. The likelihood of an individual's genotype belonging to the population where the individual has been sampled was estimated using the frequency-based method [112]. The probability that an individual was not from the local population was computed using a gamete-based Monte Carlo resampling method with 1 000 simulated individuals and a threshold of 0.01 [113]. [file 1471-2148-11-174-S1.DOC]

Additional file 1, Table S1. The geographic origins and GENECLASS assigned groups for the admixed individuals [111]; 1 represents the southern group; 2, the eastern group; 3, the northern group. The likelihood of an individual’s genotype belonging to the population where the individual has been sampled was estimated using the frequency-based method [112]. The probability that an individual was not from the local population was computed using a gamete-based Monte Carlo resampling method with 1 000 simulated individuals and a threshold of 0.01 [113].

| Sample | Geographic origin | GENECLASS highest assignment probability in three genetic lineages | GENECLASS assigned group |
| --- | --- | --- | --- |
| No.2 | DL (1) | 0.363/0/0.637 | 3 |
| No.8 | DL (1) | 0.496/0/0.504 | 3 |
| No.11 | DL (1) | 0.264/0.005/0.731 | 3 |
| No.12 | ZD (1) | 0/0.998/0.002 | 2 |
| No.27 | WX (2) | 0/0.125/0.875 | 3 |
| No.28 | WX (2) | 0/0.115/0.885 | 3 |
| No.33 | QL (2) | 0/0.457/0.543 | 3 |
| No.45 | BC (2) | 0/0.249/0.751 | 3 |
| No.46 | BC (2) | 0/0.311/0.689 | 3 |
| No.47 | BC (2) | 0.006/0.154/0.839 | 3 |
| No.49 | BC (2) | 0.028/0.332/0.641 | 3 |
| No.50 | YA (2) | 0/0.001/0.999 | 3 |
| No.51 | YA (2) | 0/0.160/0.840 | 3 |
| No.53 | YA (2) | 0/0.08/0.92 | 3 |
| No.55 | YA (2) | 0.016/0.455/0.528 | 3 |
| No.57 | MK (3) | 0.018/0.904/0.077 | 2 |
| No.58 | MK (3) | 0.96/0.04/0 | 1 |
| No.68 | MK (3) | 0.589/0/0.411 | 1 |
| No. 69 | MK (3) | 0.006/0.777/0.222 | 2 |
| No. 73 | CD (3) | 0.772/0/0.228 | 1 |
| No. 75 | CD (3) | 0.633/0/0.367 | 1 |
